# Detection rate of causal variants in severe childhood epilepsy is highest in patients with seizure onset within the first four weeks of life

**DOI:** 10.1186/s13023-018-0812-8

**Published:** 2018-05-02

**Authors:** David Staněk, Petra Laššuthová, Katalin Štěrbová, Markéta Vlčková, Jana Neupauerová, Marcela Krůtová, Pavel Seeman

**Affiliations:** 1 Department of Paediatric Neurology, DNA Laboratory, 2nd Faculty of Medicine, Charles University in Prague and University Hospital Motol, Prague, Czech Republic; 2 Department of Paediatric Neurology, 2nd Faculty of Medicine, Charles University in Prague and University Hospital Motol, Prague, Czech Republic; 3 Department of Biology and Medical Genetics, 2nd Faculty of Medicine, Charles University and University Hospital Motol, Prague, Czech Republic; 4 Department of Medical Microbiology, 2nd Faculty of Medicine, Charles University and University Hospital Motol, Prague, Czech Republic

**Keywords:** Epilepsy, Epileptic encephalopathy, Targeted gene panel testing, MPS, Phenotype, KCNQ2

## Abstract

**Background:**

Epilepsy is a heterogeneous disease with a broad phenotypic spectrum and diverse genotypes. A significant proportion of epilepsies has a genetic aetiology.

In our study, a custom designed gene panel with 112 genes known to be associated with epilepsies was used. In total, one hundred and fifty-one patients were tested (86 males / 65 females).

**Results:**

In our cohort, the highest probability for the identification of the cause of the disease was for patients with a seizure onset within the first four weeks of life (61.9% clarification rate) – about two times more than other groups. The level of statistical significance was determined using a chi-square analysis.

From 112 genes included in the panel, suspicious and rare variants were found in 53 genes (47.3%).

Among the 151 probands included in the study we identified pathogenic variants in 39 patients (25.8%), likely pathogenic variants in three patients (2%), variants of uncertain significance in 40 patients (26.5%) and likely benign variants in 69 patients (45.7%).

**Conclusion:**

Our report shows the utility of diagnostic genetic testing of severe childhood epilepsies in a large cohort of patients with a diagnostic rate of 25.8%. A gene panel can be considered as a method of choice for the detection of pathogenic variants within patients with unknown origin of early onset severe epilepsy.

**Electronic supplementary material:**

The online version of this article (10.1186/s13023-018-0812-8) contains supplementary material, which is available to authorized users.

## Background

Epilepsy is a heterogeneous disease with a broad phenotypic spectrum and diverse genotypes. A significant proportion of epilepsies has a genetic aetiology [1].

Severe childhood epilepsies are a very heterogeneous group of diseases, both clinically and genetically. Epilepsies might be inherited in an autosomal dominant fashion with mutations being often de novo, yet a good proportion of patients exhibit an autosomal recessive inheritance [1].

New methods of gene panel sequencing such as Massively Parallel Sequencing (MPS) enable a feasible approach to finding the causal variant in these patients, however the interpretation of the variants is often challenging.

The objective of our study was to identify the genetic aetiology of epilepsy in patients with severe early onset epilepsies.

## Methods

Severe epilepsy is considered to be an intractable epilepsy which usually begins in infancy and is associated with global developmental delay, cognitive dysfunction and ongoing epileptiform activity that causes further cognitive slowing and decline. Drug-resistant epilepsy is defined as a failure of two or more appropriately selected and adequately tried anticonvulsant medications to achieve seizure freedom [2, 3].

### Patients

One hundred and fifty-one unrelated patients with severe childhood epilepsy were included in the study (86 males / 65 females). In the majority of cases, the epilepsy occurred sporadically (138/152), while in the remaining 13 patients the occurrence was familial.

Probands were referred for genetic analysis over a period from March 2015 to December 2016. The patients’ legal representatives all gave informed consent and the study was approved by the local ethics committee. Brain MR imaging revealed no structural abnormalities in any of the patients. DNA samples from both parents were collected for the interpretation of variants; in two exceptional cases this was not possible.

Optional: previously tested with array CGH – to exclude chromosomal aberrations as the cause of epileptic seizures.

### Design

A custom gene panel design was created with SureDesign (SureDesign release 3.5.x, Agilent, California, USA) application. Genes were chosen according to these criteria:

1. “Known” epilepsy genes: At least two published reports describing a causal relationship between variants in the gene and epilepsy.

OR

2. At least one published report describing a causal relationship between variants in the gene and epilepsy in two or more unrelated patients.

At first, according to these criteria and a literature search, we included 97 relevant genes in the gene panel design (07/2015). Then, the second version (in 03/2016) was enriched by newly reported genes up to a final number of 112. Genes included in the designs are listed in the Additional file 1: part II.

Sequencing was performed on a MiSeq Desktop Sequencer from Illumina (Illumina, California, USA) with 2 × 150 bp sequencing kit (20 samples per run).

### Data analysis

Data were analysed by two independent software tools - NextGene (NextGENe 2.41, Softgenetics, Pennsylvania, USA) and SureCall (SureCall 3.0.3.x, Agilent, California, USA).

NextGene analysis was performed with default settings.

SureCall analysis was performed on default settings except for “SNP Read depth filter”. This value was set to value 10. The aim of this analysis was to increase the sensitivity of the whole process.

Afterwards, Alamut Batch (Alamut Batch 1.5.2, Interactive Biosoftware, Rouen, France) was used for annotating merged VCFs into a tabular file.

#### Variant evaluation

An Alamut Batch annotated file in tabular format was used as the input for evaluation. Variants were then filtered according to the following workflow:

1. Variants found in three or more patients from the same run were filtered out
2. Variants with a higher percentage (over 1%) in population databases (ExAC, 1000G) were deprioritized
3. Classification into four groups based on criteria in the annotated file (such as: ACMG classification [4], prediction programs – SIFT [5], Polyphen2 [6], Mutation taster [7], Clinvar [8], conservation, inheritance, X-linked disease). Groups were defined by pathogenicity of the variant: Pathogenic, likely pathogenic, variants of uncertain significance, benign and likely benign were grouped together.

#### CNV analysis

We used tools integrated into NextGENe for the detection of copy number variations. For the analysis, samples were compared with healthy control samples. NextGENe CNV tools perform a detection based on the Hidden Markov Model [9].

The resulting report shows INDELs in a tabular file. For precise analysis, this CNV Tool was performed against a different group of healthy controls, see Additional file 1: part III.

### Parental testing

For each pathogenic or likely pathogenic variant, Sanger sequencing and segregation analysis was performed and evaluated.

## Results

After analysis of the whole dataset, we selected 99 SNVs and one CNV for further analysis. From 112 genes included in the panel, suspicious and rare variants were found in 53 genes (47.3%).

Most frequently, variants were found in *SCN1A* (eight occurrences) and *KCNQ2* (five occurrences). All variants found in the project were then stratified into these four classes:

- Pathogenic
- Likely pathogenic
- Variants of uncertain significance (VUS)
- Benign and likely benign

**Fig. 1:**
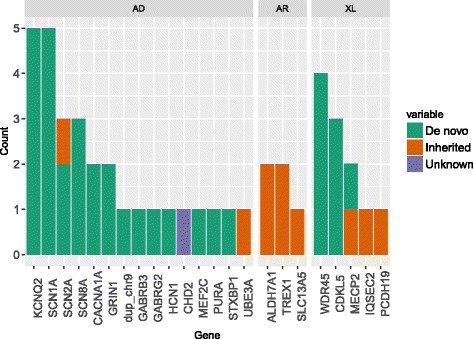
The number of pathogenic variants in individual genes stratified by inheritance mode. AD = autosomal dominant, AR = autosomal recessive, XL = X-linked. Legend: X axis: all genes Y axis: Number of pathogenic variants

For the next step, Sanger sequencing of variants, classified as Pathogenic or Likely pathogenic, was used; the total was 42 SNVs in 22 genes out of 112 in the panel (19.6%). Furthermore we identified one CNV on chr 9. (Fig. 1 and Table 1).

**Table 1:** List of variants found in cohort classified as Pathogenic or Likely Pathogenic

*Gene*	Ref Seq	DNA-level	Protein level	AD/AR	DN/INH	Prediction (SIFT, PolyPhen2,ClinVar)			ExAC all
Pathogenic AD variants									
*CACNA1A*	NM_001127221.1	c.13319826G > T		AD	DN				
*CACNA1A*	NM_001127221.1	c.2663A > T	p.Gln888Leu	AD	DN				0.0005
*dup chr9*	CNV			AD	DN				
*GABRB3*	NM_000814.5	c.841A > G	p.Thr281Ala	AD	DN	D	PD		
*GABRG2*	NM_000816.3	c.968G > A	p.Arg323Gln	AD	DN	D	PD		
*GRIN1*	NM_007327.3	c.2443G > A	p.Gly815Arg	AD	DN	D	PD	P	
*GRIN1*	NM_007327.3	c.1643G > A	p.Arg548Gln	AD	DN	T	PD		
*HCN1*	NM_021072.3	c.1189A > G	p.Ile397Leu	AD	DN	T	B		
*KCNQ2*	NM_172107.2	c.826A > C	p.Thr276Pro	AD	DN	D	B		
*KCNQ2*	NM_172107.2	c.1004C > G	p.Pro335Arg	AD	DN	D	PD		
*KCNQ2*	NM_172107.2	c.701C > T	p.Thr234Ile	AD	DN	D	PD		
*KCNQ2*	NM_172107.2	c.913_915delTTC	p.Phe305del	AD	DN				
*KCNQ2*	NM_172107.2	c.913_915delTTC	p.Phe305del	AD	DN				
*MEF2C*	NM_002397.4	c.766C > T	p.Arg256*	AD	DN				
*PURA*	NM_005859.4	c.812_814del	p.Phe271del	AD	DN				
*SCN1A*	NM_001202435.1	c.1244 T > A	p.Ile415Lys	AD	DN	D	PD		
*SCN1A*	NM_001165963.1	c.5384A > G	p.Glu1795Gly	AD	DN	D	PD		
*SCN1A*	NM_001165963.1	c.4384dup	p.Tyr1462Leufs*24	AD	DN				
*SCN1A*	NM_001165963.1	c.1178G > A	p.Arg393His	AD	DN	D	PD	P	
*SCN1A*	NM_001165963.1	c.1525C > T	p.Gln509*	AD	DN				
*SCN2A*	NM_001040142.1	c.2774 T > C	p.Met925Thr	AD	DN	D	PD		
*SCN2A*	NM_001040142.1	c.5009C > T	p.Thr1862Ile	AD	DN	T	PD		
*SCN8A*	NM_014191.3	c.4921C > G	p.Leu1641Val	AD	DN	D	PD		
*SCN8A*	NM_014191.3	c.2549G > A	p.Arg850Gln	AD	DN	D	PD	LP	
*SCN8A*	NM_014191.3	c.4850G > T	p.Arg1617Leu	AD	DN	D	PD		
*STXBP1*	NM_003165.3	c.1654 T > C	p.Cys552Arg	AD	DN	D	B		
*UBE3A*	NM_130838.1	c.1149G > C	p.Glu383Asp	AD	INH				
Pathogenic AR variants									
*ALDH7A1*	NM_001182.4	c.1318-1G > C		AR	INH				0.00041
*ALDH7A1*	NM_001182.4	c.518-14_518delinsCA		AR	INH				
*SLC13A5*	NM_177550.3	c.425C > T	p.Thr142Met	AR	INH	D	PD	P	0.00081
*TREX1*	NM_016381.3	c.10621072del	p.Leu354Phefs*22	AR	UNK				
*TREX1*	NM_016381.3	c.1072A > C	p.Thr358Pro	AR	INH	T		P	0.0016
Pathogenic X-linked variants									
*CDKL5*	NM_003159.2	c.2578C > T	p.Gln860*	XL	DN				
*CDKL5*	NM_003159.2	c.463 + 5G > A		XL	DN				
*CDKL5*	NM_003159.2	c.1247_1248del	p.Glu416Valfs*2	XL	DN			P	
*IQSEC2*	NM 001111125.2	c.3206G > C	p.Arg1069Pro	XL	INH	D	PD		
*MECP2*	NM_004992.3	c.1219_1229del	p.Asp407Glnfs*25	XL	DN				
*WDR45*	NM_007075.3	c.654del	p.Arg219Alafs*69	XL	DN				
*WDR45*	NM_007075.3	c.970_971del	p.Val324Hisfs*17	XL	DN				
*WDR45*	NM_007075.3	c.511C > T	p.Gln171*	XL	DN				
*WDR45*	NM_007075.3	c.344 + 4A > C		XL	DN				
Likely pathogenic variants									
*CHD2*	NM_001271.3	c.3782G > A	p.Trp1261*	AD	UNK				
*MECP2*	NM_004992.3	c.925C > T	p.Arg309Trp	XL	INH	D	PD	VUS	
*PCDH19*	NM_001184880.1	c.698A > G	p.Asp233Gly	XL	INH	D	PD		

Legend: Data were analysed by SureCall and NextGENe with parameters mentioned in the methods section

SIFT – D: deleterious, T: tolerated;

*PolyPhen2 PD* probably damaging, *B* benign, *PoD* possible damaging;

*ClinVar – VUS* Variant of uncertain significance, *P* pathogenic;

*AR* autosomal recessive, *AD* autosomal dominant, *XL* X-linked, *INH* inherited, *DN* de novo

The rest of the variants (VUS and Benign) are summarized in Additional file 1: part IV.

**Fig. 2:**
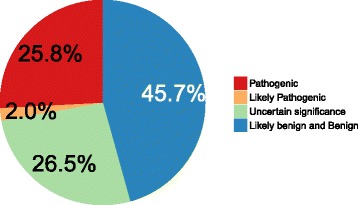
Patients classified by detected variants There were 25.8% of probands with pathogenic variants (39/151), 2% probands with likely pathogenic variants (3/151), 26.5% with variants of uncertain significance (40/151), and 45.7% of probands without suspicious variants (69/151). If the proband had more than one variant, the “most pathogenic” variant was used to designate pathogenicity

Among the 151 probands included in the study, we identified pathogenic variants in 39 patients (25.8%), likely pathogenic variants in three patients (2%), variants of uncertain significance in 40 patients (26.5%) and likely benign variants in 69 patients (45.7%) (Fig. 2). Two patients were carriers of two pathogenic variants – one was a compound heterozygote for two variants in trans in the *ALDH7A1*, the second was a compound heterozygote for two variants in trans in the *TREX1*.

### Inheritance patterns

Our results showed that the majority of pathogenic or likely pathogenic variants were found in genes that follow autosomal dominant pattern of inheritance (27 SNVs in 14 genes), also CNV on chr9 was found to be acting with AD inheritance. Another 5% were found in genes associated with autosomal recessive inheritance (five SNVs in three genes), and finally 24% were found in genes with X-linked inheritance (11 SNVs in five genes).

For 40 patients, out of 42 classified with pathogenic or likely pathogenic variants, DNA samples from both parents were available for segregation analysis by Sanger sequencing.

In 34 patients, including 33 with SNVs and one with CNV, these variants arose de novo. These de novo variants were found in 15 genes and CNV was found in chromosome 9.

Nine variants in seven genes (detected in 7 patients) were inherited.

In two cases only one parental sample was available. In one of these cases the detected variant was inherited from the mother.

Information about maternal or paternal inheritance of inherited (and unknown) variants are available in Additional file 1: part V.

### The distribution of the age at seizure onset and age at inclusion into the study

The distribution of the age at seizure onset and age at inclusion into the study is described in the Additional file 1: part I. The information was gathered from the patient’s documentation. The median age at seizure onset of the whole group was 14.5 months; the first quartile was 4 months and the third quartile was 36 months. The median age at inclusion into study was 93 months; the first quartile was 49.5 months and the third quartile was 169 months.

### The probability of finding the pathogenic variant in relation to age at seizure onset

The gene panel testing indicated that the highest probability for finding the cause of epilepsy was in the cohort of patients with the earliest onset of seizures, i.e. within the first four weeks of life – 61.9% clarification rate (13/21 patients).

**Fig. 3:**
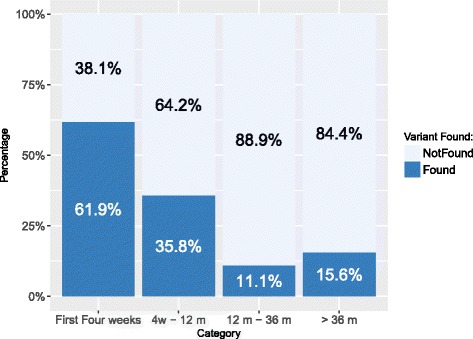
Classification based on age at the onset of seizures: Legend: X axis: probands were divided into 4 groups by the age at the first seizure – the first four weeks of life, four weeks to 12 months of age, 12 months to 36 months years of age and older than 36 months., Y axis: Percentage of variants (not found) – pathogenic and likely pathogenic variants were marked as “variant found”

In other age groups the clarification rate was lower: 35.8% (19/53) in the group of patients with first seizure between four weeks and 12 months of age; 11.1% (5/45) in the group with first seizure between 12 months and 36 months of age; and 15.6% in patients with first seizure after the age of 36 months (5/32) (Fig. 3).

The dataset was analysed using the Chi squared statistic and the results were statistically significant (*p* = 0.000052; the result is significant at *p* < 0.05). Moreover, we always compared two groups with the Fisher’s exact test and significant relationships between the age at onset and clarification rate were observed for group 1 (<four weeks) and group 2 (4 weeks – 12 months) *p* = 0.0673; group 1 and group 3(12 months – 36 months) *p* = 0.0001; group 1 and group 4 (> 36 months) *p* = 0.0009. However, no significant difference was observed when comparing group 3 vs. group 4 (p = value 0.7330).

## Discussion

We were able to identify the cause of severe childhood epilepsy in 25.8% of patients from our cohort and this finding concurs with previously published reports [1, 2]. From the 112 genes in the panel, pathogenic or likely pathogenic variants occurred in 22 of them.

MPS gene panel testing enables the testing of a large number of genes in parallel with very high coverage and low costs. More supportive information is presented in Additional file 1: part VI.

Over 80% of the pathogenic variants arose de novo, as they were not present in the parental samples (confirmed by Sanger sequencing).

### Special cases

The first case was a patient with a CHD2 variant, where the father’s sample was not available. The second patient, with two heterozygous variants in gene TREX1, has one variant inherited from his/her mother (p.Thr358Pro) while the other variant is of unknown inheritance, as the father’s sample was not available. A female proband with Angelman syndrome has a variant in gene UBE3A inherited from her healthy mother. This is caused by imprinting, when the proband’s maternal allele is active and the paternal is not expressed. The mother inherited the variant from her father.

### Comparison with previously published reports about epilepsy gene panels

**Fig. 4:**
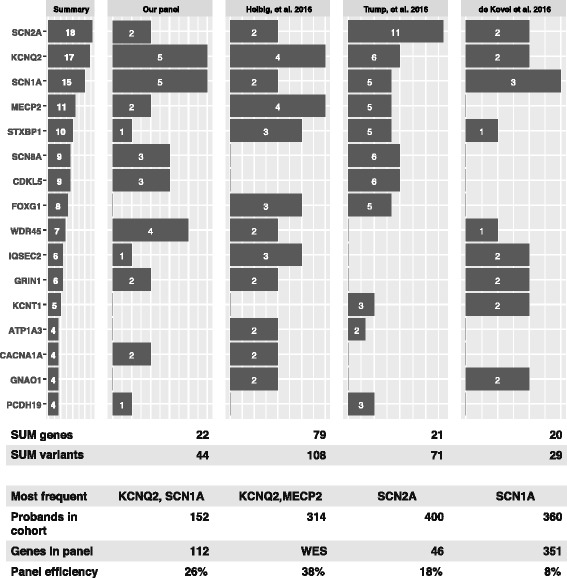
Results of our gene panel analysis were compared with three other published studies that focused on targeted sequencing in patients with epilepsy. For comparison, the following sources were used: [9–11]

In order to assess the sensitivity and specificity of our approach, we compared our data with previously published reports. The analysis is presented in Fig. 4.

In agreement with the study by Helbig, et al. [10], the diagnostic rate was approximately 30%. Moreover, the vast majority of the pathogenic variants in currently known genes are de novo*.* Our results are very similar to those shown by Trump, et al. [11] (94%). According the study by Kovel, et al. [12], in which the authors adopted a very different approach and designed a large panel consisting of 351 genes, the diagnostic efficiency of their panel did not increase and was much lower than the expected 30%.

Designing a large panel can help to find some rare variants which other panels cannot reveal; but it also involves higher costs, lower coverage, lower read depth and more difficult interpretations of the more variants that are multiplied by each sample. Based on our experience, we would recommend the design of a panel with approximately 100 well selected genes.

### Age at seizure onset

Our results show that the probability of finding the pathogenic variant is the highest in patients with the earliest age at seizure onset (results in Fig. 3). Among our patients, the detection rate for pathogenic or likely pathogenic variant found in a group with seizure onset during the first four weeks of life was 61.9%.

In the groups where the onset of seizure was later, the number of pathogenic or likely pathogenic variants was found to be significantly lower. In a group where the age of seizure onset was between four weeks to 12 months it was 35.8%; between 12 months and 36 months, 11.1%; and after 36 months, 21.7%.

In effect, the earlier the phenotype is manifest then the chances of finding a pathogenic variant are significantly higher. This is shown in Fig. 3.

This trend has also been described previously by Helbig, et al. [10].

#### CNV testing

In our cohort two CNVs were found. The first classified as Pathogenic (on chr 9) for epilepsy and the second classified as VUS. These were detected using NextGENe CNV comparison tool based on Hidden Markov Model Results and were further confirmed by an Array CGH.

## Conclusion

Our study has proven that MPS gene panel is a powerful tool for the DNA diagnosis of severe MRI negative childhood epilepsies. Today, a gene panel is an optimal method for the identification of pathogenic variants in highly heterogeneous disorders such as the genetically determined disorders including severe childhood epilepsies. In a cohort of 151 patients, we were able to identify the cause of epilepsy in 27.8% of patients (39 patients with pathogenic variant and three with likely pathogenic variants).

## Additional file

Additional file 1: Part I Age distribution among patients, first box plot is age of seizure onset, second age of inclusion into study. Part II List off all genes included in panel. Part III Process of CNV analysis. Part IV List of variants of uncertain significance or likely benign found in our cohort. Part V. Part VI Advantages of the gene panel testing. (DOCX 150 kb)

## Abbreviations

AD: Autosomal dominant
AR: Autosomal recessive
CNV: Copy number variation
MPS: Massively parallel sequencing
SNV: Single nucleotide variant
VUS: Variant of uncertain significance
WES: Whole exome sequencing
