# Supplementary material for: Detection rate of causal variants in severe childhood epilepsy is highest in patients with seizure onset within the first four weeks of life
Source: Orphanet J Rare Dis. 2018 May 2;13:71. doi: 10.1186/s13023-018-0812-8 (PMC5932755; doi:10.1186/s13023-018-0812-8)
Supplement: Supplementary file 1 — Part I Age distribution among patients, first box plot is age of seizure onset, second age of inclusion into study. Part II List off all genes included in panel. Part III Process of CNV analysis. Part IV List of variants of uncertain significance or likely benign found in our cohort. Part V. Part VI Advantages of the gene panel testing. (DOCX 150 kb) [file 13023_2018_812_MOESM1_ESM.docx]

# Additional file

## Additional file part I

Additional file I Age distribution among patients, first box plot is age of seizure onset, second age of inclusion into study

|  | Age of onset [months] | Age of inclusion [months] |
| --- | --- | --- |
| Mean | 24.4 | 121 |
| Standard deviation | 30 | 109.85 |
| First quartile | 4 | 49.5 |
| Second quartile / median | 14.5 | 93 |
| Third quartile | 36 | 169 |

## Additional file part II

| *ADAR* | *CNTNAP2* | *GPHN* | *KCTD7* | *PNPO* | *SCN8A* | *SZT2* |
| --- | --- | --- | --- | --- | --- | --- |
| *ADCK3* | *DCX* | *GRIN1* | *MBD5* | *POLG* | *SCN9A* | *TBC1D24* |
| *ADSL* | *DNM1* | *GRIN2A* | *MECP2* | *PRIMA1* | *SLC12A5* | *TCF4* |
| *ALDH7A1* | *DOCK7* | *GRIN2B* | *MEF2C* | *PRRT2* | *SLC13A5* | *TDP2* |
| *ALG13* | *EEF1A2* | *HCN1* | *MFSD8* | *PSEN1* | *SLC19A3* | *TPP1* |
| *AMT* | *FASN* | *HDAC4* | *MOCS1* | *PURA* | *SLC25A22* | *TREX1* |
| *AP4S1* | *FLNA* | *HNRNPU* | *MOCS2* | *QARS* | *SLC2A1* | *TSC1* |
| *ARHGEF9* | *FOLR1* | *CHD2* | *MTHFR* | *RNASEH2A* | *SLC35A2* | *TSC2* |
| *ARX* | *FOXG1* | *IQSEC2* | *MTOR* | *RNASEH2B* | *SLC9A6* | *UBE3A* |
| *ASAH1* | *GABBR2* | *KCNA2* | *NEDD4L* | *RNASEH2C* | *SPTAN1* | *WDR45* |
| *ATP1A3* | *GABRA1* | *KCNB1* | *NRXN1* | *ROGDI* | *SRGAP2* | *ZEB2* |
| *BRAT1* | *GABRB3* | *KCNC1* | *PANK2* | *RYR3* | *SRPX2* |  |
| *C10ORF2* | *GABRG2* | *KCNH5* | *PCDH19* | *SAMHD1* | *ST3GAL3* |  |
| *CACNA1A* | *GAMT* | *KCNJ10* | *PHF6* | *SCN1A* | *ST3GAL5* |  |
| *CASK* | *GCSH* | *KCNQ2* | *PIGQ* | *SCN1B* | *STXBP1* |  |
| *CDKL5* | *GLDC* | *KCNQ3* | *PLCB1* | *SCN2A* | *SYN1* |  |
| *CLCN4* | *GNAO1* | *KCNT1* | *PNKP* | *SCN4A* | *SYNGAP1* |  |

Additional file II List off all genes included in panel

Above is a list of all genes included in the gene panels. Genes with yellow background were added to the second version of the panel (03/2016). Gene SCN9A (with green background) was not included in the newer version of the panel.

## Additional file part III


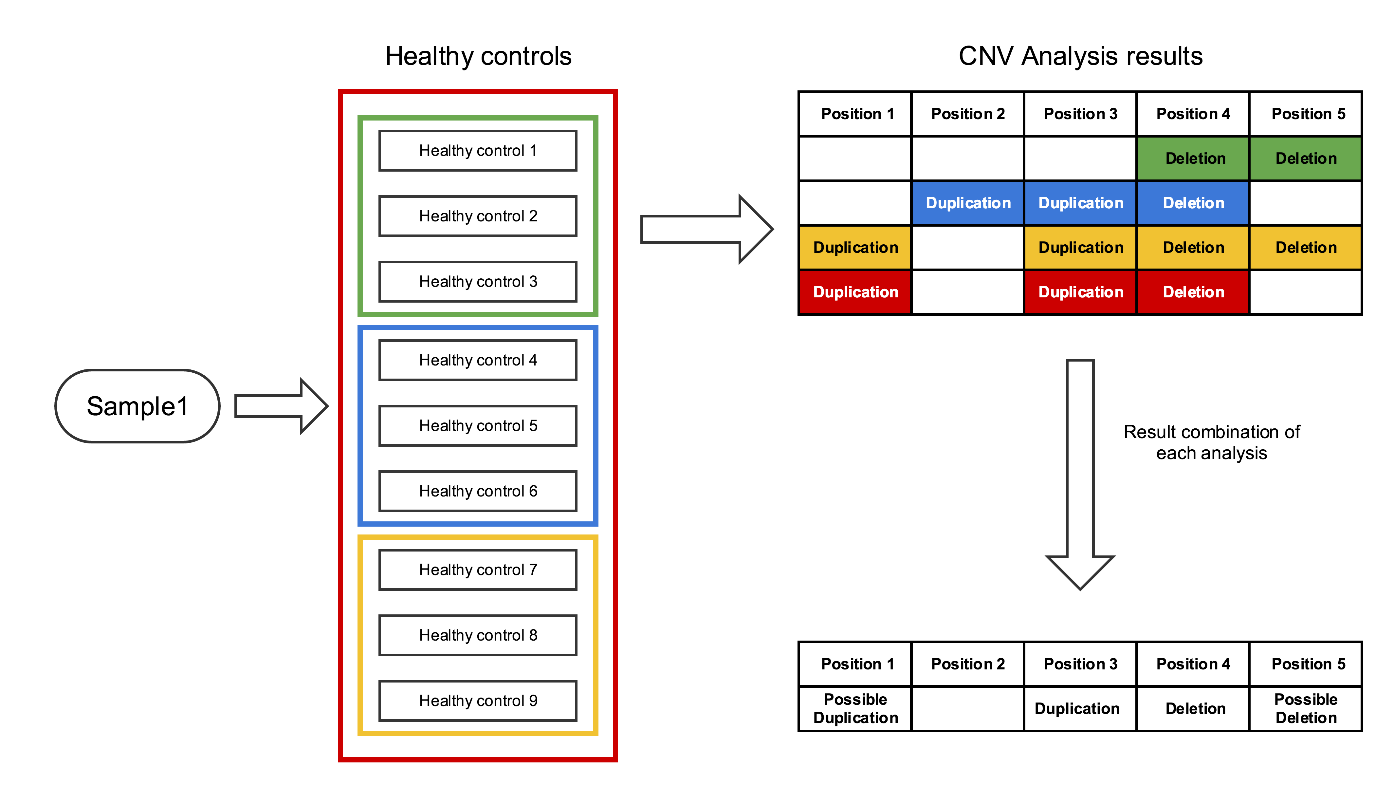


Additional file III Process of CNV analysis

For each sample we used nine healthy controls (sex matched) divided into three groups. CNV analysis was performed with a sample against each group and against all healthy controls. Results of these analyses were then combined together and only matching CNVs were used for further consideration.

## Additional file part IV

| ***Gene*** | **Ref Seq** | **Variation at DNA-level** | **Variation at protein level** | **Prediction  (SIFT, PolyPhen2,ClinVar)** | | | **AD/AR** | **DN/ INH** |
| --- | --- | --- | --- | --- | --- | --- | --- | --- |
| *ATP1A3* | NM_152296.4 | c.829G>A | p.Glu277Lys | D | PD | P | AD | DN |
| *ATP1A3* | NM_152296.4 | c.1472A>G | p.Asn491Ser | T | B |  | AD |  |
| *CACNA1A* | NM_001127221.1 | c.6811G>A | p.Val2271Ile | T | B |  | AD |  |
| *FASN* | NM_004104.4 | c.1465G>A | p.Glu489Lys | T | B |  | AD |  |
| *FASN* | NM_004104.4 | c.532G>A | p.Gly178Arg | D | PD |  | AD | INH |
| *GABRG2* | NM_198903.2 | c.1032A>C | p.Arg344Ser | D | PD | P | AD | INH |
| *GRIN2B* | NM_000834.3 | c.3873G>T | p.Gln1291His | T | B |  | AD |  |
| *CHD2* | NM_001271.3 | c.2293T>A | p.Cys756Ser | D | PoD |  | AD |  |
| *KCNQ3* | NM_004519.3 | c.2481G>A | p.Met827Ile | T | B |  | AD |  |
| *KCNT1* | NM_020822.2 | c.457C>T | p.Tyr153His |  |  |  | AD |  |
| *NEDD4L* | NM_015277.5 | c.126T>C | p.Asp42Glu |  |  |  | AD |  |
| *NEDD4L* | NM_015277.5 | c.568C>T | p.Leu190Phe | T | PoD |  | AD |  |
| *SCN1A* | NM_001165963.1 | c.80G>C | p.Arg27Thr | T | B | VUS | AD |  |
| *SCN1A* | NM_001165963.1 | c.4001G>A | p.Arg1334Lys | D | PoD |  | AD |  |
| *SCN4A* | NM_000334.4 | c.2211C>G | p.Cys737Trp | D | PD |  | AD |  |
| *SCN4A* | NM_000334.4 | c.739G>A | p.Val247Met | T | PD |  | AD |  |
| *TREX1* | NM_016381.3 | c.23_24del | p.Gln8Argfs*9 |  |  |  | AD |  |
| *TSC2* | NM_000548.3 | c.5008C>G | p.His1670Asp | T | B |  | AD | INH |
| *TSC2* | NM_000548.3 | c.4761C>G | p.Cys1587Trp | D | PD |  | AD |  |
| *KCNJ10* | NM_002241.4 | c.1049G>A | p.Gly350Glu | T | B |  | AR | INH |
| *MFSD8* | NM_152778.2 | c.1340C>T | p.Pro447Leu |  | PD |  | AR | INH |
| *RNASEH2A* | NM_006397.2 | c.635A>G | p.Asn212Ser | T | B |  | AR |  |
| *RNASEH2A* | NM_006397.2 | c.715C>T | p.Arg239Cys | D | B |  | AR |  |
| *RNASEH2B* | NM_024570.3 | c.859G>T | p.Ala287Ser | T | PD |  | AR |  |
| *ST3GAL5* | NM_003896.3 | c.353del | p.Lys118Argfs*70 |  |  |  | AR |  |
| *TBC1D24* | NM_001199107.1 | c.1008del | p.His336Glnfs*12 |  |  | P | AR | INH |
| *FLNA* | NM_001456.3 | c.5740G>A | p.Val1914Met | D | PD |  | XL |  |
| *FLNA* | NM_001456.3 | c.5213C>T | p.Pro1738Leu | T | B |  | XL |  |
| *FLNA* | NM_001456.3 | c.2515A>G | p.Thr839Ala | T | B |  | XL |  |
| *ASAH1* | NM_177924.4 | c.536C>T | p.Thr179Ile | D |  |  |  |  |
| *BRAT1* | NM_152743.3 | c.1432C>G | p.Leu478Val | D | PoD |  |  |  |
| *BRAT1* | NM_152743.3 | c.358C>T | p.Arg120Cys | D | PoD |  |  |  |
| *EEF1A2* | NM_001958.3 | c.789del | p.Val264Trpfs*15 |  |  |  |  |  |
| *MTHFR* | NM_005957.4 | c.1162C>T | p.Arg388Cys | D | PD |  |  |  |
| *MTOR* | NM_004958.3 | c.2558A>G | p.Tyr853Cys | D | PD |  |  |  |
| *PANK2* | NM_153638.2 | c.1168A>G | p.Ile390Val | D | PoD |  |  |  |
| *PIGA* | NM_002641.3 | c.290T>C | p.Met97Thr | T | PD |  |  |  |
| *PLCB1* | NM_015192.3 | c.2995G>A | p.Ala999Thr | T | B |  |  |  |
| *QARS* | NM_005051.1 | c.1426G>A | p.Val476Ile | T | PoD |  |  |  |
| *ROGDI* | NM_024589.2 | c.320A>G | p.Gln107Arg | T | PoD |  |  |  |
| *RYR3* | NM_001036.4 | c.10931T>C | p.Met3644Thr | D | B |  |  |  |
| *RYR3* | NM_001036.4 | c.3970A>G | p.Thr1324Ala | T | B |  |  |  |
| *RYR3* | NM_001036.4 | c.6452C>T | p.Ala2151Val | D | PoD |  |  |  |
| *RYR3* | NM_001036.4 | c.1437+7C>T |  |  |  |  |  |  |
| *SLC2A1* | NM_006216.2 | c.1220C>T | p.Ala407Val |  |  |  |  |  |
| *SLC6A1* | NM_003042.3 | c.1531G>A | p.Val511Met | D | PD |  |  |  |
| *SZT2* | NM_015284.3 | c.429C>T | p.Ile143Ile |  |  |  |  |  |
| *GRIN2B* | NM_000834.3 | c.2172-6G>A |  |  |  |  | AD |  |
| *KCNA2* | NM_004974.3 | c.17G>A | p.Gly6Glu | T | B |  | AD |  |
| *KCNT1* | NM_020822.2 | c.2452A>G | p.Ile818Val | T | B |  | AD |  |
| *NEDD4L* | NM_001144967.2 | c.698C>T | p.Ser233Leu | T | B |  | AD |  |
| *SCN1A* | NM_001165963.1 | c.3521C>G | p.Thr1174Ser | T | B | VUS | AD |  |
| *SCN4A* | NM_000334.4 | c.113G>A | p.Arg38Gln | D | B |  | AD |  |
| *TSC2* | NM_000548.3 | c.5246G>A | p.Arg1749Gln | D | PD |  | AD |  |
| *PNKP* | NM_007254.3 | c.1029+2T>C |  |  |  |  | AR |  |
| *FLNA* | NM_001456.3 | c.4985C>T | p.Thr1662Met | T | PoD |  | XL |  |

Additional file IV List of variants of uncertain signicifance or likely benign found in our cohort

Legend:

data were analysed by SureCall and NextGENe with parameters mentioned in methods

SIFT – D: deleterious, T:tolerated;

PolyPhen2 PD: probably damaging, B: benign, PoD: possibly damaging;

ClinVar – VUS: Variant of uncertain significance;

ACMG class – P: Pathogenic, LP: Likely pathogenic, VUS: Variant of uncertain significance;

AR = autosomal recessive, AD = autosomal dominant, XL = X-linked; INH = inherited; DN = de novo

## Additional file part V

| ***Gene*** | **Ref Seq** | **DNA-level** | **Protein level** | **AD/AR** | **DN/INH** | **Variant inherited from:** |
| --- | --- | --- | --- | --- | --- | --- |
| *UBE3A* | NM_130838.1 | c.1149G>C | p.Glu383Asp | AD | INH | Mother and father |
| *ALDH7A1* | NM_001182.4 | c.1318-1G>C |  | AR | INH | Mother and father |
| *ALDH7A1* | NM_001182.4 | c.518-14_518delinsCA |  | AR | INH | Mother and father |
| *SLC13A5* | NM_177550.3 | c.425C>T | p.Thr142Met | AR | INH | Mother and father |
| *TREX1* | NM_016381.3 | c.10621072del | p.Leu354Phefs*22 | AR | UNK | Not in mother’s sample, father’s sample not available |
| *TREX1* | NM_016381.3 | c.1072A>C | p.Thr358Pro | AR | INH | Mother, father’s sample not available |
| *IQSEC2* | NM 001111125.2 | c.3206G>C | p.Arg1069Pro | XL | INH | Mother, not in father’s sample |
| *CHD2* | NM_001271.3 | c.3782G>A | p.Trp1261* | AD | UNK | Not in mother’s sample, father’s sample not available |
| *MECP2* | NM_004992.3 | c.925C>T | p.Arg309Trp | XL | INH | Mother, not in father’s sample |
| *PCDH19* | NM_001184880.1 | c.698A>G | p.Asp233Gly | XL | INH | Mother, not in father’s sample |

## Additional file part VI

### Advantages of the gene panel testing

Gene panel MPS enables the testing of large number of genes in parallel with very high coverage and low costs. Gene panels have been recently widely used for groups of highly heterogeneous disorders.

Regarding coverage, gene panel testing is truly the best choice and has several advantages over WES. Compared to WES, gene panel testing provides almost 100% coverage over many otherwise uncovered regions. Gene panel testing is thus the method of choice for diagnostic testing. It is currently the most precise approach reaching the reliability of Sanger sequencing which is seen as the gold standard yet with WES such coverage is not possible.

In comparison, WES with a very good design, has many regions uncovered (1 007 801 bp uncovered of 50 390 061 bp). On the other hand gene panel testing with coverage of 99.99% has only 48 bp uncovered (of 482 648 bp).

On the other hand, Sanger sequencing has some drawbacks, namely related to primer mismatch. Moreover, Sanger sequencing of all target genes is laborious and needless with current technologies. When considering diagnostic testing of a target region, gene panel testing is currently the most reliable and most effective method.

From an ethical point of view, we think gene panel testing is the best choice in diagnostic genetic testing. This is based on recommendation from ESHG^[[1]](#footnote-1)^, which supports the idea of observing only a part of the genome that has already been connected to the phenotype. By using this approach, research can focus on the area of interest and ensure maximum patient data safety. WES represents a different approach providing a wider insight to the human genome. This “hunting for everything” approach can sometimes be more beneficial and can reveal some additional useful information, but from our point of view, is not appropriate as the first test.

1. Van El CG, Cornel MC, Borry P, et al. Whole-genome sequencing in health care: Recommendations of the European Society of Human Genetics. *European Journal of Human Genetics*. 2013;21(6):580-584. doi:10.1038/ejhg.2013.46. [↑](#footnote-ref-1)
